# Collectanea Medica: Consisting of Anecdotes, Facts, Extracts, Illustrations, &c.

**Published:** 1825-09

**Authors:** 


### COLLECTANEA MEDICA:

CONSISTING OF

### ANECDOTES, FACTS, EXTRACTS, ILLUSTRATIONS, &c.

*Relating to the History or the Art of Medicine, and the Collateral Sciences.*

---

Floriferus, ut apes, in saltibus omnia libant,
Omnia nos, itidem, depascimur aurea dicta.

---

### ART. I.—*Extracts from* MR. SHAW'S "*Further Observations on the Lateral or Serpentine Curvature of the Spine.*"

#### *On the Means generally used with the intention of curing a Stoop.*

As a stoop frequently accompanies a slight distortion, and as the means commonly used to counteract it are not only ineffectual, but even tend to increase the bend, it will not be out of place here to inquire into the methods of treating it, especially as the one which I have to propose assists in removing a slight degree of lateral distortion.

When the chest and the head fall forward, the most common method of trying to correct the stoop, is to put on some instrument by which the shoulders and the head are held back. To operate upon the shoulders, the common back-collar is applied; and, to hold back the head, a riband is brought over the forehead, and fastened to the collar.

While these instruments are kept on, the figure looks straight, though stiff and constrained; but, the moment they are taken off, both the head and the shoulders fall more forward than before their application. Many examples of the bad effect of artificially supporting the head might be offered. The following example, although it is to be observed in the figure of a horse, is very demonstrative. When the rein (called the bearing-rein,) by which the head of a carriage-horse is reared up, with the intention of giving him a showy figure, is loosened, the head immediately falls forward, and the neck, instead of having the fine arch that is so much admired, droops between the shoulders. Looking to this effect, we should at first be inclined to condemn the practice followed by horse-dealers, of reining up the head of a young horse in the stable, by means of the apparatus called a dumb jockey. But, on examining into this mode of fixing the head, it will be found to operate on a very different principle from the bearing-rein. Instead of a simple bit, such as the horse in harness can lean his head upon without suffering any pain, a bit calculated to teaze and fret is put into the young horse's mouth. To relieve himself from the irritation produced by this, and which is increased by the constant pull of the elastic piece of iron to which the rein is fastened, he curls up his neck, and thus brings all the muscles of the back of the neck into strong action, instead of allowing their power to be superseded by the artificial support afforded by the bearing-rein to the horse in harness.*

---

* When the Russians wish to give a horse high action in trotting, they accustom

Many different contrivances, but all acting nearly on the same principle as the bearing-rein, have been proposed as means for obliging a girl to keep her head erect.

There is one mode which, to a person ignorant of anatomy, seems to be particularly well adapted for this purpose, but it is in fact more objectionable than the plan of tying the head back with a riband. A piece of lead, of some pounds weight, is slung over the back in such a way that it must be supported by a riband put around the head. Although this contrivance prevents the head for a time from falling forwards, its bad effects may be demonstrated. When the weight is on, the muscles of the back of the spine are passive, while those on the fore-part of the neck are necessarily brought into action to prevent the head from being pulled too far back; and this is easily proved: if we put the fingers on the sternal portions of the sterno-cleido muscle, which, with the small muscles on the fore-part of the throat, pull the head forwards, we shall feel them tense and in action; and, to show still further the increased activity of the muscles on the fore-part, and the passive condition of those of the back, we have only to raise the weight when the girl is not aware of our doing so, the head will then be immediately poked forwards.

I lately saw an ingenious piece of mechanism intended to hold the head back, by producing the same effect as the weight suspended by the riband: it is recommended by Mr. Bampfield. But it is scarcely necessary to add, that the objections made to the use of the weight, on the ground of the anterior muscles being excited by it, while those of the back of the neck are passive, are equally applicable to this contrivance.

We have many opportunities of observing the incorrectness of the principle on which all similar plans for the cure of a stoop have been founded. For instance, porters who carry burthens on the back, by the assistance of a band round the forehead, always stoop; while those who carry baskets before them, suspended by a band round the back of the neck, are peculiarly erect. But the most remarkable example of the effect from the head being pulled back by a weight hung behind, is the condition of the women who carry salt in the streets of Edinburgh, —for they may be recognised as much by their miserable sardonic grin, which is caused by the constant excitement of the platysma myoides muscle, as by their stoop.

Such results may, perhaps, be thought scarcely worthy of notice; but the very worst consequences may ensue from any system of treatment where a constant resistance to the muscles of the fore-part of the neck is kept up. A gentleman had for many years worn one of the collars invented by Mr. Chesher. By using this machine, two very bad effects were produced: the muscles of the back were so weakened as to be rendered incapable of supporting the vertebral column, while those on the fore-part of the

---

him, while young, to wear very heavy shoes on the fore feet. We can now, perhaps, understand how this produces the desired effect: the resistance to be overcome necessarily increases the strength of certain muscles, and hence, when shoes of the common size are put on, the horse will lift his feet higher than one which has not been subjected to this discipline. Since writing this, I have been told that opera dancers practise with lead weights on their shoes.

neck were so disproportionately increased in strength, by the constant resistance opposed to them by the strap passing from the suspending rod under the chin, that, whenever the strap was loosened, the chin was forcibly drawn towards the chest. As the muscles of the back part of the neck did not offer any counteracting resistance, the windpipe was now pressed down, or almost doubled on itself. As soon as this took place, (and it was almost immediate on the attempt to sit up without the collar,) the patient was seized with such a sense of suffocation as to be obliged to throw himself on his back. As he was able to breathe with ease while he lay on his back, his advisers were led farther into error, and believed that it was the weight of the head which pressed down the windpipe. To counteract this pressure, various contrivances had been proposed to support the head. Indeed, the patient himself was so convinced, from what he had heard, that it was the weight of the head which pressed down the windpipe, and so alarmed had he become from the certainty of having a fit of suffocation when the head was left unsupported, that I had much difficulty in persuading him to believe that, if the head could be made heavier, the sense of suffocation would be relieved. I at length induced him, although he submitted with great dread of the consequence, to allow me to place about fourteen pounds of shot on the top of his head. He was very much alarmed ; but it was highly gratifying to witness his surprise and pleasure in finding that, instead of his head being weighed down, he could support it, and could breathe with ease while in the upright posture. The principle on which I proceeded was this:—The muscles of the back part of the neck had been brought into such a state, that their ordinary stimulus was not sufficient to excite them to the action necessary to counteract the efforts of those on the fore-part of the neck, which had been evidently increased in strength. The placing a weight on a certain spot on the head formed an additional stimulus to the muscles of the back part of the neck ; a fact which the reader may prove by an experiment on himself.

By proceeding on this principle, by combining a variety of exercises, and by gradually diminishing the weight carried on the head, I had very soon the pleasure of seeing my patient walking and sitting in a state of great comfort, without being obliged to use any artificial support.

I have since used nearly the same means, and with considerable success, in the case of a patient who was suffering from a paralytic affection of some of the muscles of the back part of the neck. I wish I had thought of it while attending a lady, who had a very peculiar nervous affection, which gave her the feeling of being about to shake her head off.

It is well known that instruments, similar to that represented in the wood-cut,* support almost the whole weight of the head and shoulders by the strap which passes under the chin. It must also have been observed that the wearer very frequently pushes down the head against the chin-strap. In this way, the muscles on the fore-part necessarily

---

* The instrument represented is a piece of iron fastened to the back, and curving over the head, with a strap passing under the chin.

become stronger, while those of the back, being deprived of their natural stimulus to action, in consequence of the rod superseding their office, become diminished in power. Even were there no change in the degree of strength in the muscles on the fore-part, the head would naturally fall, if the support afforded by the chin-strap were removed; but, as these muscles are increased in power, while those of the back are diminished, the head must not only fall, but even be pulled down.

However, although the collars and the lead weight, as they are generally used, are not only inefficacious, but even hurtful, they may occasionally be useful in keeping the head in a certain position, after it has been brought to it by such exercises as tend to strengthen those muscles of the back which support the shoulders and head. But so completely do I differ from the opinions commonly entertained as to the means of counteracting an habitual stoop, that I would almost recommend the position of a tailor sitting on his shop-board, as more advantageous than the systems generally followed. This at first appears ridiculous; but the manner in which a tailor holds his body when he walks, proves that there is something in his habits which tends to the correction of a stoop; for he is quite a caricature of a strutting erect figure, especially in the way he bends in his loins and carries his head.

The peculiarity of the tailor's gait proceeds, in a certain degree, from the bent position in which he sits: but this explanation is not at first satisfactory, since it may be observed that other tradesmen, who also stoop while at work, generally have their head inclined forwards, and have also a distinct and habitual bend in the neck; such, especially, is the condition of persons who sit at a table and stoop forwards,—as watch-makers, engravers, &c. It is not difficult to explain the cause of the difference, and the inquiry will assist in directing us to the principles which we ought to recollect in our operations upon the spine.

In the sitting position of the tailor, the head hangs so low, and so complete an arch is formed between it and the pelvis, that the muscles of the spine are called into strong action to support the head; the necessary consequence of this is, that these muscles become even unnaturally strong, or at least so strong as to predominate over those by which the spine is pulled forward. But the bent position is not the only cause of increase in the strength of the muscles, for it depends also on the exercise given by frequently jerking the head backwards. In those who stoop from the middle of the body, as in writing or working at a table, the muscles of the spine are not called into action; for, while the head is in this position, it rests, or is supported by the ligament of the neck. The ligament being thus kept constantly on the stretch, becomes lengthened, instead of being made more contractile, as muscles would be; and hence the stoop is increased. When this is combined with the consequences of the want of muscular action, the deeper ligaments which bind the upper vertebræ gradually yield: if the operation of these causes continues for a certain time, the bones and cartilages themselves become altered in shape, and consequently an almost irremediable stoop is produced.

This view derives confirmation from what may be observed in the shape of the tailors in some parts of Germany, who, instead of having the erect figures of London tailors, are quite bent. On inquiring into the cause, we find that, instead of sitting as tailors do in this country, a hole is cut in the table, and a seat is placed within it; so that their position, while working, becomes nearly the same as that of persons who sit at a table.

It may, perhaps, be objected that labourers, and especially the vine-dressers in France, are remarkable for the complete arch which their body forms, although they bend while at work as much as the tailor does. This may also be explained; for, in the labourer, the bend is produced by the pelvis rolling on the head of the thigh-bones, while, in a person sitting as a tailor, the pelvis continues nearly fixed, and the bend is in the vertebræ on the pelvis.

The erect figure of the Turk, perhaps, comes from the manner of sitting which is common among eastern nations; but the heavy turban, and the spice-box slung from the back of the neck, may account in a great measure for the fine figures of the Turkish Jews who frequent the streets of London.

We may even take the shoe-maker as an example of the effect of a particular manner of sitting, and of frequently using the muscles of the shoulders. He is also a little in caricature, but he carries himself better than the tailor, and the cause is obvious. The tailor's figure is very erect, but the right shoulder is generally a little higher or larger than the left, from the constant exercise given to the right arm, while the left rests upon the knee: this inequality of the shoulders is not observed in the shoe-maker, because he not only uses both arms equally, but the muscles by which the scapulæ are supported become so strong by the habit of jerking back his elbows while he works, that his shoulders always appear more braced back than those of any other class of persons: indeed, so characteristic are the figures of tailors and shoemakers, that they may be easily distinguished in a crowd.

I have mentioned these circumstances, because they afford familiar examples of the principles on which we ought to proceed in endeavouring to correct deformities; but it would be ridiculous to propose the position either of the tailor or of the shoe-maker, as the best adapted to correct a stoop or falling forward of the shoulders; though, in very young patients, I have found it expedient to put all their playthings on the ground, and to recommend such games as will induce them, while sitting, to bend the body and raise the head alternately. In patients farther advanced, much benefit has been derived from the use of an instrument which was planned with the intention of bringing the muscles of the back part of the neck into action. I have since simplified it very much, and contrived to fix it to the back of a chair, so that its use is attended with scarcely any inconvenience or annoyance to the patient. This contrivance is useful not only in strengthening the muscles of the back, but, when combined with a proper support, it admits of a young lady writing, drawing, or playing on the pianoforte, without any risk of increasing the lateral curvature of the spine.

### *On the Treatment of Contracted Joints.*

In the preceding volumes, it has been shown whence it happens that rubbers and shampooers are sometimes successful in the treatment of distortions and contractions of the limbs, when surgeons have failed. It is there stated that their occasional success seems to arise from pressure, thumbing, &c. calling into action parts which, from lying long dormant, have become feeble and useless. The cases where this practice is most likely to be attended with benefit are those of stiff and contracted joints, after rheumatism, or any chronic inflammation. But, to do good even in such cases, great perseverance is necessary; and a degree of boldness, which *à priori* we should almost consider dangerous. The professed rubber proceeds in a much more violent manner than those who know the structure of the parts would venture upon, without some previous evidence of the practice being harmless, although, indeed, this violence may be one cause of the rubber's success. But such bold practitioners may occasionally do harm, as they are seldom capable of distinguishing between the contractions attending the acute inflammations of joints, and those which are the consequences of chronic affections; and are also inattentive to the distinctions of constitution, and the possibility of rousing a scrofulous action. However, instances of bad effects from their mode of practice seem to be rarer than we might expect; but we may not hear of all that occur; for, although every instance where a quack is successful is blazoned about, parents are so far ashamed of entrusting their children to the care of ignorant persons, that they always endeavour to conceal any mischief that has been done.

When a surgeon, for the first time, witnesses the operations of a professed rubber, he is a little startled at the violence of his operations, and is surprised at the manner delicate patients bear them. Such were my own impressions at first; but having, about eight years ago, had frequent opportunities of seeing a famous rubber at work, and having witnessed the result of his treatment in several cases, I was so satisfied that, if judiciously combined with other modes, it might not only be safe, but of the greatest use, that I have since been in the habit of ordering the women, whom I employ on these occasions, to rub and shampoo with a degree of violence which, to some practitioners, might appear almost unwarrantable.

It is scarcely necessary to state, that the nature of the case must be carefully investigated before any mode of treatment is determined upon, and that, from whatever cause the motion of a joint may have been lost, we should be very cautious in our first attempts to restore it. If the bones be anchylosed, our labour will be in vain, and the attempt to move the joint may be dangerous; but, if the bones can be moved in the slightest degree, we may calculate on doing good, for the stiffness may proceed only from inflammation changing the natural secretions of the sheaths of the tendons, or from adhesions having taken place between those parts. By rubbing, and gentle attempts at motion, the cellular membrane, by which the tendons and sheaths are united, may be loosened and extended, the contracted ligaments may be lengthened, and the muscles resume their natural structure and functions. Liniments and oils of different kinds are generally employed by rubbers, with the intention of *suppling the joints.* The use of them is certainly attended with advantage, for a great deal of friction is necessary to cause their absorption, on which the rubber supposes the *charm* of the treatment depends. They are also useful in removing any remaining inflammatory state of the joint, or in preventing its return.

But, although rubbing, shampooing, and a variety of exercises, are most useful, and occasionally successful, they should be considered as only part of the plan of treatment; for the position in which a contracted joint is kept, is as important to its cure as the occasional relaxation and exercise. But so much harm has been done by instruments, that parents, and even many practitioners, seem to have a complete dread of them. They are, however, often absolutely necessary; for it will be found as difficult to remedy a contracted and distorted limb, without the assistance of some means to support and preserve it in a certain position, as it is to cure a distortion of the spine merely by exercise. In every case of contraction, the cure will be at least much expedited by any means, however simple, by which the limb may be preserved, during the time it is not exercised, in the improved condition into which it has been brought by the shampooing, &c. Two essential points are gained by keeping the limb in a right position. The alteration in the form of the heads of the bones, which is always, to a certain degree, the consequence of the contracted state of the joints, will not be so likely to increase; for the bones are no longer allowed to remain in the position which produced the change, and the muscles and ligaments that have been contracted will actually grow longer if kept extended. This fact is very important in practice.

Proceeding on these views, I have always insisted on the application of some means to prevent the contraction after the limb has been shampooed, and have been particularly careful to keep the limbs in a proper position during the night. Happily there are few cases where this cannot be done, and the means of doing it are generally so simple, that even irritable and restless children forget the restraint after one or two nights.

If the limb be kept constantly encased in a machine, and no exercise whatever permitted, its muscles will waste, so that, at the expiration of the period that was promised to be sufficient to effect a cure, the limb will be found incapable of supporting the weight of the body. It may, moreover, be stated, that a limb, from being thus rendered dormant, seems to be more than usually liable to be hurt by pressure; and hence we often see large and galling sores produced by the iron supports.

It is from these obviously bad effects, that shampooing, &c. have been of late used so much more than instruments; and certainly, if either system of treatment is to be used singly, the latter is better than the former. But it is surprising that the two modes have not been more frequently combined, as the one assists the other very much, while either, when used singly, is rarely attended with success.

It might be expected that I should detail the manner of treating each case; but the forms of contracted limbs are so various, and the causes from which they proceed are so many, that it is not possible to do more than point out the general principle of the treatment. Its application to the individual cases must be left to the ingenuity of the practitioner.

The most difficult cases we meet with are those where the contraction of the limbs is combined with a certain degree of palsy, both of the body and mind. In some instances, the mind is in such a condition as to render all our attempts abortive; but, unless there is absolute imbecility, we ought not to give up any case as hopeless, for the mind in children often improves in the same ratio with the progress or increase in the bodily powers, and it is well known that nothing tends so much to give command over the muscles as repeated efforts to acquire it. One grand difficulty in the treatment of such cases is to excite the child to put these muscles into action which we wish particularly to exercise. To effect this, we have not only to contrive such modes of performing exercises as will bring certain muscles into play, but we have to combine them with some amusement* that will induce the child to put them into action; for, however useful shampooing, rubbing, &c. may be, their effect upon the muscular system is as nothing compared to that of voluntary exertion. The importance of a child acquiring a voluntary power over its muscles, (and which it may do by practice,) is proved by the fact that a child may be unable to walk, although all the muscles necessary to the action are sufficiently powerful, as is shown by the resistance they offer when we pull against them.†

---

* Children in this state vary so much in disposition, that the same amusement will not do for all; but I have generally found that a noisy toy, connected with the exercising pullies, was the most effectual inducement to work.

† The application of this principle will be found very useful in cases where, after a paralytic attack, one arm continues affected: the patient should take up small articles with the weak hand, and endeavour to place them in certain determined positions, as in making chess-moves, &c. If she should have, previously to the attack, been able to play on the pianoforte, practising on it will tend very much to restore the voluntary power over the muscles of the arm.

